# Development and validation of a recommended checklist for assessment of surgical videos quality: the LAParoscopic surgery Video Educational GuidelineS (LAP-VEGaS) video assessment tool

**DOI:** 10.1007/s00464-020-07517-4

**Published:** 2020-04-06

**Authors:** Valerio Celentano, Neil Smart, Ronan A. Cahill, Antonino Spinelli, Mariano Cesare Giglio, John McGrath, Andreas Obermair, Gianluca Pellino, Hirotoshi Hasegawa, Pawanindra Lal, Laura Lorenzon, Nicola De Angelis, Luigi Boni, Sharmila Gupta, John P. Griffith, Austin G. Acheson, Tom D. Cecil, Mark G. Coleman

**Affiliations:** 1 Department of Colorectal Surgery, Portsmouth Hospitals NHS Trust, Portsmouth, UK; 2 University of Portsmouth, Portsmouth, UK; 3 Exeter Health Services, Research Unit, Royal Devon & Exeter Hospital, Exeter, UK; 4 Colorectal Unit, Mater Misericordiae University Hospital, Dublin, Ireland; 5 Section of Surgery and Surgical Specialities, School of Medicine, University College Dublin, Dublin, Ireland; 6 Humanitas Clinical Research Center - IRCCS, via Manzoni 56, 20089 Rozzano, Milan, Italy; 7 Department of Biomedical Sciences, Humanitas University, via Rita Levi Montalcini 4, 20090 Pieve Emanuele, Milan, Italy; 8 Department of Clinical Medicine and Surgery, Federico II University of Naples, Naples, Italy; 9 Royal Devon and Exeter NHS Trust, Exeter, UK; 10 University of Exeter Medical School, Exeter, UK; 11 Queensland Centre for Gynaecological Cancer, Royal Brisbane and Womens Hospital, Brisbane, Australia; 12 Faculty of Medicine, UQCCR, The University of Queensland, Herston, Brisbane, Australia; 13 Department of Medical, Surgical, Neurologic, Metabolic and Ageing Sciences, Luigi Vanvitelli University, Naples, Italy; 14 Department of Surgery, Tokyo Dental College, Ichikawa General Hospital, Ichikawa City, Japan; 15 Maulana Azad Medical College, University of Delhi, New Delhi, India; 16 Lok Nayak Hospital, New Delhi, India; 17 Fondazione Policlinico Universitario Agostino Gemelli IRCCS, Rome, Italy; 18 Unit of Digestive and HPB Surgery, CARE Department, Henri Mondor Hospital and University Paris-Est, Creteil, France; 19 Department of General and Emergency Surgery, IRCCS, Fondazione Ca’ GrandaPoliclinico Hospital, Milan, Italy; 20 University of Milan, Milan, Italy; 21 Colchester Hospital NHS Foundation Trust, Colchester, UK; 22 Bradford Teaching Hospitals NHS Foundation Trust, Bradford, UK; 23 National Institute for Health Research (NIHR) Nottingham Biomedical Research Centre, Nottingham University Hospitals NHS Trust and University of Nottingham, Nottingham, UK; 24 Peritoneal Malignancy Institute, Basingstoke, UK; 25 Peninsula School of Medicine & Dentistry, Plymouth University, Plymouth, UK; 26 Department of Colorectal Surgery, University Hospitals Plymouth NHS Trust, Plymouth, UK

**Keywords:** Laparoscopic surgery, Video assessment tool, Guidelines, Minimally invasive surgery, Surgical training

## Abstract

**Introduction:**

There has been a constant increase in the number of published surgical videos with preference for open-access sources, but the proportion of videos undergoing peer-review prior to publication has markedly decreased, raising questions over quality of the educational content presented. The aim of this study was the development and validation of a standard framework for the appraisal of surgical videos submitted for presentation and publication, the LAParoscopic surgery Video Educational GuidelineS (LAP-VEGaS) video assessment tool.

**Methods:**

An international committee identified items for inclusion in the LAP-VEGaS video assessment tool and finalised the marking score utilising Delphi methodology. The tool was finally validated by anonymous evaluation of selected videos by a group of validators not involved in the tool development.

**Results:**

9 items were included in the LAP-VEGaS video assessment tool, with every item scoring from 0 (item not presented in the video) to 2 (item extensively presented in the video), with a total marking score ranging from 0 to 18. The LAP-VEGaS video assessment tool resulted highly accurate in identifying and selecting videos for acceptance for conference presentation and publication, with high level of internal consistency and generalisability.

**Conclusions:**

We propose that peer review in adherence to the LAP-VEGaS video assessment tool could enhance the overall quality of published video outputs.

**Graphic Abstract:**

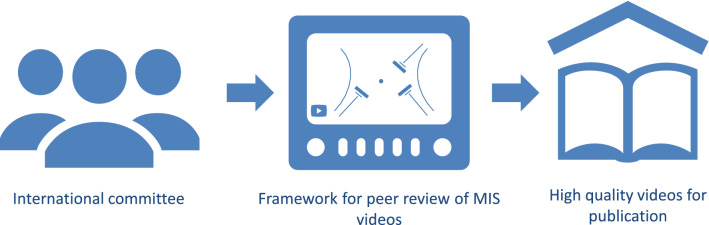

**Electronic supplementary material:**

The online version of this article (10.1007/s00464-020-07517-4) contains supplementary material, which is available to authorized users.

Minimally invasive surgery platforms facilitate the production of audio–visual educational materials with the video recording of the procedure providing viewers with crucial information concerning the anatomy and the different steps and challenges of the surgical procedure from the operating surgeon’s point of view. Surgical trainers consider online videos as a useful teaching aid [1] that maximises trainees’ learning and skill development given the backdrop of time constraints and productivity demands [2], whilst there is widespread adoption of live surgery sessions and video-based presentations at surgical conferences [3]. In fact, there has been a constant increase in the number of published surgical videos per year, [4] with preference for free access sources. Controversially, the proportion of videos undergoing peer-review prior to publication has been decreasing, raising questions over quality of the educational content provided [4], likely reflecting the difficulties on achieving a prompt and good-quality peer review [5]. Trainees value highly informative videos detailing patients’ characteristics and surgical outcomes, and integrated with supplementary educational content such as screenshots and diagrams to aid the understanding of anatomical landmarks and subdivision of the procedure into modular steps [6]. Based on these premises the LAP-VEGaS guidelines (LAParoscopic surgery Video Educational GuidelineS), a recommended checklist for production of educational surgical videos, were developed by an international, multispecialty, joint trainers–trainees committee with the aim to reduce the gap between surgeons’ expectations and online resources’ quality [7], to improve the educational quality of the video outputs when used for the scope of training. However, the question of how effectively and objectively assess videos submitted as educational or publication material remains unanswered as no template exists to date for critical appraisal and review of submitted video outputs.

The aim of this study was the development and validation of a standard framework for the appraisal of surgical videos submitted for presentation and publication, the LAP-VEGaS video assessment tool.

## Methods

An international consensus committee was established and tasked with the development of an assessment tool for surgical videos submitted for conference presentation or journal publication. Committee members were selected based on the previously published research on minimally invasive surgery training delivery [8] and evaluation [9], surgical videos availability [4] and use [6], laparoscopic surgery video guidelines development [7]. The choice of the members of the committee was conceived to include 15 participants representative of worldwide surgical trainers in different specialties, including at least one representative from general surgery, lower and upper gastrointestinal surgery, gynaecology and urology. The checklist was developed in agreement with The Appraisal of Guidelines Research and Evaluation Instrument II (Agree II, https://www.agreetrust.org/agree-ii).

The first phase of the study consisted in identifying the items for inclusion in the LAP-VEGaS video assessment tool. The steering committee was responsible for the selection of the different topics to be discussed and items were finalised after discussion through e-mails, teleconferences, and face-to-face meetings with semi-structured interviews. The discussion focused on skill domains that are important for competency assessment and on the structure of a useful video assessment marking sheet, taking into account the need for a readily applicable and easy to use marking tool, preferring items assessing the required standards for acceptance of a video for publication or conference presentation. Items for inclusion were identified from the previously published LAP-VEGaS guidelines [7] (appendix 1) and the Laparoscopic Competence Assessment Tool (LCAT) [10]. The LCAT is a task-specific marking sheet for the assessment of technical surgical skills in laparoscopic surgery designed to assess the surgeon’s performance by watching a live, live-streamed or recorded operation. The LCAT was not designed to assess videos’ educational content, but it is a score based on safety and effectiveness of the surgery demonstrated, developed by dividing the procedure into four different tasks with each task having 4 different items with a pass mark defined by receiver operating characteristics (ROC) curve analysis and validated in a previous study [11].

These items were revised by all members of the committee and based on the results of the discussion; the steering committee prepared a Delphi questionnaire, which committee members voted upon during phase II of the study utilising an electronic survey tool (Enalyzer, Denmark, www.enalyzer.com). The Delphi method is a widely accepted technique for reaching a consensus amongst a panel of experts [12]. The experts respond anonymously to at least two rounds of a questionnaire; providing a revised statement and/or explanation when voting against a statement [13]. An a priori threshold of ≥ 80% affirmative votes was needed for acceptance. Feedback on the items that did not reach 80% agreement was revised by the steering committee after the first round and statements were reviewed and resubmitted for voting.

Finally, to test the validity of the marking score, during phase 3 of the study, the steering committee selected laparoscopic videos for assessment using the newly developed LAP-VEGaS video assessment tool. Videos freely available on open-access websites not requiring a subscription fee were preferred as previously reported as the most accessed resources [6]. Videos were selected by the steering committee to allow widespread presence of content demonstrating general, hepatobiliary, gynaecology, urology, lower and upper gastrointestinal surgery procedures. Videos already presented at conferences or published on journals were excluded, if this was clearly evident from the video content or narration. The videos were anonymously evaluated by committee members and by laparoscopic surgeons not involved in the LAP-VEGaS guidelines and video assessment tool development (“validators”), according to their specialties. The resulting scores were compared for consistency and inter-observer agreement, whilst the assessment on the perceived quality of the video was performed by asking to the video reviewers if they would have recommended the video to a peer/trainee and if they would have accepted the video for a publication or podium presentation, with the use of dichotomous and 5-point Likert scale questions (Table 1).

**Table 1:** 5-point Likert scale on recommendations of the video to a peer/trainee and on acceptance of the video for publication or podium presentation

	Items	1	2	3	4	5
1	I would recommend this video to a peer/trainee	**☐ **strongly disagree	**☐** disagree	**☐** neither agree/disagree	**☐** agree	**☐** strongly agree
2	The video is of satisfactory quality for a presentation/publication	**☐** strongly disagree	**☐** disagree	**☐** neither agree/disagree	**☐** agree	**☐** strongly agree
3	Overall quality of the video	**☐** very poor	**☐** poor	**☐** average	**☐** good	**☐** very good
4	Overall educational content of the video	**☐** very poor	**☐** poor	**☐** average	**☐** good	**☐** very good
5	How long it took to complete the marking score (only time needed for completion of the score, not video time)	**☐** < 1 min	**☐** 1–2 min	**☐** 2–3 min	**☐** 3–4 min	**☐** > 4 min
6	How satisfied are you with using the score?	**☐** very unsatisfied	**☐** unsatisfied	**☐** neither unsatisfied/satisfied	**☐** satisfied	**☐** very satisfied
7	I would you use the LAP-VEGaS marking score again?	**☐** strongly disagree	**☐** disagree	**☐** neither agree/disagree	**☐** agree	**☐** strongly agree
8	The items of the LAP-VEGaS score help differentiating educational/non-educational–good-quality/poor-quality videos	**☐** strongly disagree	**☐** disagree	**☐** neither agree/disagree	**☐** agree	**☐** strongly agree

### Statistical analysis

Concurrent validity of the video assessment tool was tested against the expert decision on recommending the video for publication or conference presentation. For such analysis, Receiving Operator Characteristics (ROC) curves analysis was used. The Area under the ROC curve was used as an estimator of the test concurrent validity, with values superior to 0.9 indicating high validity [14]. The Youden Index was used to identify a cut-off value maximising sensitivity and specificity values [15].

Internal test consistency (i.e. across-items consistency) was estimated by Cronbach’s Alpha and using the Spearman–Brown Prophecy Coefficient, to make this analysis independent from the numbers of items. Each item’s impact on the whole tool reliability was measured as changes in Cronbach’s alpha following item deletion.

Inter-observer reliability was estimated by the analysis of the Intra-class Correlation Coefficient (ICC) and Cronbach’s alpha. ICC was estimated along with its 95% confidence interval, based on the mean rating and using a one-way random model, since each video was rated by a different set of observers. Intra-observer reliability, which estimated the test consistency over the time, was analysed by the test and re-test technique and the Pearson’s r correlation coefficient was used (with * r *> 80 indicating good reliability).

The generalisability of the LAP-VEGaS video assessment tool’s results was further tested according to the generalisability theory. The generalisability (G) coefficient was estimated according to a two-facet nested design [16] with the two facets being represented by tool items and reviewers. A decision (D) study was conducted to define the number of assessors needed to maximise the G-coefficient.

*p* values ≤ 0.05 were considered as statistically significant. All statistical analysis was performed using IBM SPSS Statistics for Windows, version 25.0 (IBM Corp., Armonk, NY, USA).

## Results

### Delphi consensus and LAP-VEGaS video assessment tool development

Phase I terminated with the steering committee preparing a Delphi questionnaire of 14 statements (Appendix 2).

All 15 committee members completed both the first and the second round of the Delphi questionnaire with results presented in Table 2, with 9 items selected for inclusion in the LAP-VEGaS video assessment tool, with every item scoring from 0 (item not presented in the video) to 2 (item extensively presented in the video), with a total marking score ranging from 0 to 18 (Table 3).

**Table 2:** Results of the Delphi process for inclusion of items in the LAP-VEGaS video assessment tool

Nr	Item description	Mean score	Standard deviation
1	Authors and Institution information. Title of the video including name of the procedure and pathology treated	4.8	0.1
2	Formal presentation of the case, including age, sex, American society of Anaesthesiologist Score (ASA), Body Mass Index (BMI), indication for surgery, comorbidities and history of previous surgery. Anonymised relevant imaging is presented	4.6	0.4
3	Position of patients, access ports, extraction site and surgical team	4.8	0.2
4	The surgical procedure is presented in a standardised step-by-step fashion	4.7	0.3
5	The intraoperative findings are clearly demonstrated, with constant reference to the anatomy	4.8	0.2
6	Relevant outcomes of the procedure are presented, including operating time, length of hospital stay and postoperative morbidity^b^	4.4	0.5
7	Histopathology assessment of the specimen is presented, supported by pictures of the specimen(s)^b^	3.5	0.6
8	Additional educational content is included. (Diagrams, photos, snapshots and tables used to demonstrate anatomical landmarks, relevant or unexpected finding)	4.0	0.7
9	Audio/written commentary in English language is provided	4.4	0.6
10	The image quality is appropriate with constant clear view of the operating field and appropriate camera angle. Video speed is appropriate	4.8	0.2
11	The video demonstrates an unusual case or management of intraoperative complications^a^	3.3	0.9
12	The procedure demonstrates competent use of dominant and nondominant hand with appropriate degree of traction and safe use of grasping and dissecting instruments^a^	3.2	0.9
13	The procedure demonstrates appropriate speed and economy of movements, finishing one step before starting the next and avoiding rough tissue handling and unnecessary movements^a^	3.2	0.9
14	The video is recorded full length or with minimal editing^a^	2.4	0.9

Steering committee members answered the question “This item should be included in the LAP-VEGaS marking sheet”: 1. Strongly disagree, 2. Disagree, 3. Neither agree or disagree, 4. Agree, 5. Strongly agree

^a^Items not reaching ≥ 80% consensus even following the second round of voting were not included

^b^Following round one, items 6 and 7 were collated into one single item and reached a 4.7 ± 0.2 agreement in round 2 of the Delphi process

**Table 3:** LAP-VEGaS video assessment tool

	Item description	Not presented (0)	Presented, partially (+ 1)	Presented, completely (+ 2)
1	Authors and Institution information. Title of the video including name of the procedure and pathology treated	**☐**	**☐**	**☐**
2	Formal presentation of the case, including patient details and imaging, indication for surgery, comorbidities and previous surgery. Patient anonymity is maintained	**☐**	**☐**	**☐**
3	Position of patient, access ports, extraction site and surgical team	**☐**	**☐**	**☐**
4	The surgical procedure is presented in a standardised step by step fashion	**☐**	**☐**	**☐**
5	The intraoperative findings are clearly demonstrated, with constant reference to the anatomy	**☐**	**☐**	**☐**
6	Relevant outcomes of the procedure are presented, including operating time, postoperative morbidity and histology when appropriate	**☐**	**☐**	**☐**
7	Additional graphic aid is included such as diagrams, snapshots and photos to demonstrate anatomical landmarks, relevant or unexpected finding, or to present additional educational content	**☐**	**☐**	**☐**
8	Audio/written commentary in English language is provided	**☐**	**☐**	**☐**
9	The image quality is appropriate with constant clear view of the operating field. The video is fluent with appropriate speed	**☐**	**☐**	**☐**

### Video assessment

**Fig. 1:**
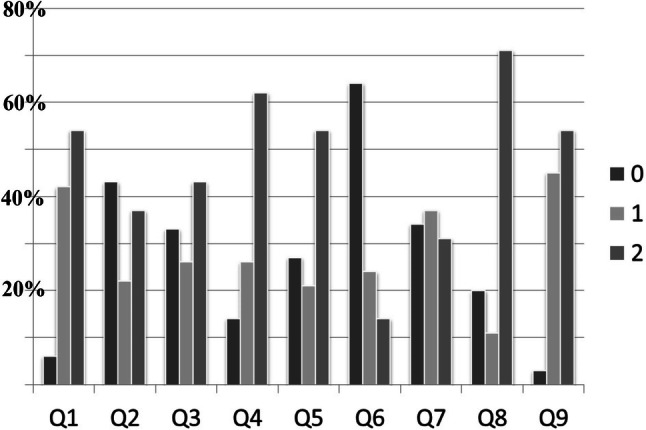
Distribution of scores for each of the 9 items of the video assessment tool. Q1–Q9: Items of the assessment tool. 0 Item not presented. 1 Item partially presented. 2 Item extensively presented

The newly developed LAP-VEGaS video assessment tool was used for assessment of 102 free access videos, which were evaluated by at least 2 reviewers and 2 validators. There was an excellent agreement amongst different reviewers in the decision to recommend the video for conference presentation and journal publication (*K* = 0.87, *p* < 0.001). The distribution of scores for each of the 9 items of the assessment tool is presented in Fig. 1.

The validators reported that the median time for completion of the LAP-VEGaS score was 1 min to 2 min. Moreover, there was a high level of satisfaction with the use of the LAP-VEGaS video assessment tool amongst the validators, reporting a median of 4.5 and 5 to the 5 point Likert scale question “Overall Satisfaction” and “How likely are you to use this tool again”, respectively.

### Reliability and generalisability analysis

The LAP-VEGaS video assessment tool showed good internal consistency (Cronbach’s alpha 0.851, Spearman–Brown coefficient 0.903). No item exclusion was found to significantly improve the test reliability (maximum Cronbach’s alpha improvement 0.006).

Strong inter-observer reliability was found amongst the different reviewers (Cronbach’s alpha 0.978; ICC 0.976, 95% CI 0.943–0.991, *p* < 0.001) and when comparing scores between experts and validators (Cronbach’s alpha 0.928; ICC 0.929, 95% CI 0.842–0.969, *p* < 0.001).

**Fig. 2:**
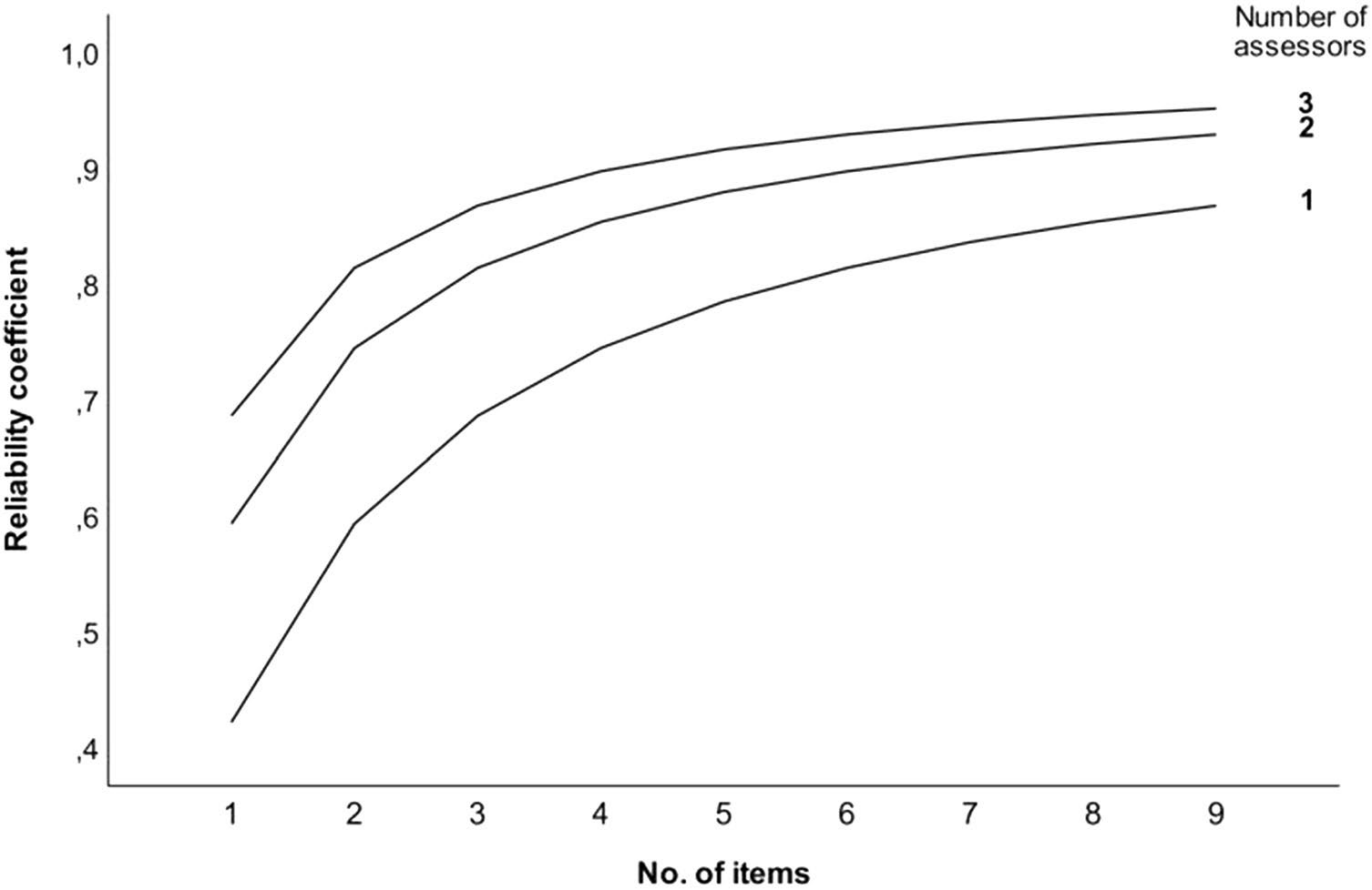
Reliability analysis. The D-study showed that test reliability was maximised when 3 assessors scored the video (G-coefficient 0.952), although scores by 2 assessors ensured optimal and similar results (G-coefficient 0.929)

The video assessment tool demonstrated a high level of generalisability (G-coefficient 0.952), Fig. 2.

### Validity analysis

The LAP-VEGaS video assessment tool resulted highly accurate in identifying and selecting videos for acceptance for conference presentation and publication (AUC 0.939, 95% CI 0.897–0.980, *p* < 0.001). The Area under the ROC curve demonstrated that a total score of 11 or higher at the LAP-VEGaS video assessment tool correlated with recommended acceptance for publication or podium presentation, with a sensitivity of 94% and specificity of 73%, whilst a score of 12 or higher had a sensitivity of 84% and a specificity of 84%.

## Discussion

We present the LAP-VEGaS video assessment tool, which has been developed and validated through consensus of surgeons across different specialties to provide a framework for peer review of minimally invasive surgery videos submitted for presentation and publication. Peer review of submitted videos aims to improve the quality and educational content of the video outputs and the LAP-VEGaS video assessment tool aims to facilitate and standardise this process. Interestingly, there is currently no standard accreditation or regulation for medical videos as training resources [17]. The HONCode [18] is a code of conduct for medical and health websites, but this applies to all online content and is not specific for audio–visual material. The EQUATOR (Enhancing the QUAlity and Transparency Of health Research) Network (https://www.equator-network.org) lists reporting guidelines which have been developed, mainly driven by the insufficient quality of published reports [19]. Some of these are internationally endorsed guidelines such as CONSORT Statement for randomised controlled trials [20], STROBE for observational studies in epidemiology [21] and PRISMA for systematic reviews and meta-analyses [22]. The previously published LAP-VEGaS guidelines [7] and the hereby presented LAP-VEGaS video assessment tool provide reference standards not only for preparation of videos for submission, but also for peer assessment prior to publication. The LAP-VEGaS video assessment tool has been developed according to a rigorous methodology involving selection of items for inclusion in the marking score and agreement on items by an international multispecialty committee utilising Delphi methodology. The distribution of the newly developed LAP-VEGaS video assessment tool to a group of users, completely independent from the steering committee, finally validated the score for video evaluation and recommendation for publication or conference presentation. The score demonstrated a sensibility of 94% for a mark of 11 or higher, with its validity as a screening tool for videos submitted for publication confirmed by the ease of use reported by the reviewers, who spent an average of 1 to 2 min to complete the score. Our results support that peer review of videos using the LAP-VEGaS video assessment tool should be performed by at least two assessors addressing all nine items of the marking score. Nevertheless, it is important to consider that the acceptability of a video submitted for publication still remains a subjective process, which also depends on variables that cannot be captured by the LAP-VEGaS video assessment tool, such for instance the readership or audience, and the current availability of videos showing the same procedure.

Reporting guidelines facilitate good research and their use is indirectly influencing the quality of future research, as being open about the study shortcomings when reporting one study can influence the conduct of the next study. Constructive criticism based on the LAP-VEGaS video assessment tool could ensure the credibility of the resource and the safety of the procedure presented, with an expected resultant improvement in the quality of the educational videos available on the World Wide Web.

The LAP-VEGaS video assessment tool provides a basic framework that standardises and facilitates video content evaluation when peer-reviewing videos submitted for publication or presentation, despite recognising that the cognitive load of the procedure presented is only one of several key elements in video-based learning in surgery [23]. Teamwork and communication are paramount for safe and effective performance and have not been explored in this video assessment tool, which focus on surgeon’s technical skills [24]. An additional limitation of our assessment tool is that it was developed for assessment of video content presenting a stepwise procedure, and it does not apply to all educational surgical video outputs, such for instance basic skills’ training or videos demonstrating a single step of a procedure, which may not need such extensive clinical detail.

It is important to acknowledge that there are minimal data available in the published literature to base this consensus video assessment tool development and validation on high-quality evidence. Nevertheless, the Delphi process with pre-set objectives is an accepted methodology to reduce the risk of individual opinions prevailing, and the selected co-authors of these practice guidelines have previously reported on the topic of surgical videos’ availability, quality [4], content standardisation [7], and use by surgeons in training [6].

Moreover, the LAP-VEGaS video assessment tool may generate widespread availability of videos demonstrating an uncomplicated procedure [25], resulting in publication bias [26] the same way that research with a positive result is more likely to be published than inconclusive or negative studies, as researchers are often hesitant to submit a report when the results do not reach statistical significance [27]. To allow wider acceptance of the LAP-VEGaS video assessment tool, this should now be evaluated by surgical societies across different specialties, conference committees and medical journals with the aim to improve and standardise the quality of the shared content by increasing the number of videos undergoing structured peer-review facilitated by the newly developed marking score. We propose that peer review in adherence to the LAP-VEGaS video assessment tool could help improve the overall quality of published video outputs.

## Electronic supplementary material

Below is the link to the electronic supplementary material.

Supplementary file1 (DOCX 18 kb)

Supplementary file2 (DOCX 19 kb)

## References

[1] Abdelsattar JM, Pandian TK, Finnesgard EJ (2015). Do you see what I see? How we use video as an adjunct to general surgery resident education. J Surg Educ.

[2] Gorin MA, Kava BR, Leveillee RJ (2011). Video demonstrations as an intraoperative teaching aid for surgical assistants. Eur Urol.

[3] Rocco B, Grasso AAC, De Lorenzis E (2018). Live surgery: highly educational or harmful?. World J Urol.

[4] Celentano V, Browning M, Hitchins C (2017). Training value of laparoscopic colorectal videos on the World Wide Web: a pilot study on the educational quality of laparoscopic right hemicolectomy videos. Surg Endosc.

[5] Stahel PF, Moore EE (2014). Peer review for biomedical publications: we can improve the system. BMC Med.

[6] Celentano V, Smart N, Cahill RA (2018). Use of laparoscopic videos amongst surgical trainees in the United Kingdom. Surgeon.

[7] Celentano V, Smart N, Cahill R (2018). LAP-VEGaS practice guidelines for reporting of educational videos in laparoscopic surgery: a joint trainers and trainees consensus statement. Ann Surg.

[8] Coleman M, Rockall T (2013). Teaching of laparoscopic surgery colorectal. The Lapco model. Cir Esp.

[9] Miskovic D, Wyles SM, Carter F (2011). Development, validation and implementation of a monitoring tool for training in laparoscopic colorectal surgery in the English National Training Program. Surg Endosc.

[10] Mackenzie H, Ni M, Miskovic D (2015). Clinical validity of consultant technical skills assessment in the English National Training Programme for Laparoscopic Colorectal Surgery. Br J Surg.

[11] Miskovic D, Ni M, Wyles SM (2013). Is competency assessment at the specialist level achievable? A study for the national training programme in laparoscopic colorectal surgery in England. Ann Surg.

[12] Linstone HA, Turoff M (1975). The Delphi Method Techniques and Applications.

[13] Varela-Ruiz M, Díaz-Bravo L, García-Durán R (2012). Description and uses of the Delphi method for research in the healthcare area. Inv Ed Med.

[14] Swets JA (1988). Measuring the accuracy of diagnostic systems. Science.

[15] Youden WJ (1950). Index for rating diagnostic tests. Cancer.

[16] Brennan RL (2001). Generalizability theory.

[17] Langerman A, Grantcharov TP (2017). Are we ready for our close-up? Why and how we must embrace video in the OR. Ann Surg.

[18] Health On the Net Foundation. The HON Code of Conduct for medical and health Web sites (HONcode). https://www.healthonnet.org/. Accessed 1 July 2019

[19] Simera DG, Altman DM (2008). Guidelines for reporting health research: the EQUATOR Network's survey of guideline authors. PLoS Med.

[20] Moher D, Hopewell S, Schulz KF (2010). CONSORT 2010 explanation and elaboration: updated guidelines for reporting parallel group randomised trials. BMJ.

[21] von Elm E, Altman DG, Egger M (2007). The strengthening the reporting of observational studies in epidemiology (STROBE) statement: guidelines for reporting observational studies. Lancet.

[22] Liberati A, Altman DG, Tetzlaff J (2009). The PRISMA statement for reporting systematic reviews and meta-analyses of studies that evaluate healthcare interventions: explanation and elaboration. J Clin Epidemiol.

[23] Ritter EM (2018). Invited editorial LAP-VEGaS practice guidelines for video-based education in surgery: content is just the beginning. Ann Surg.

[24] Sgarbura O, Vasilescu C (2010). The decisive role of the patient-side surgeon in robotic surgery. Surg Endosc.

[25] Mahendran B, Caiazzo A (2019). Transanal total mesorectal excision (TaTME): are we doing it for the right indication? An assessment of the external validity of published online video resources. Int J Colorectal Dis.

[26] Mahid SS, Qadan M, Hornung CA, Galandiuk S (2008). Assessment of publication bias for the surgeon scientist. Br J Surg.

[27] Dickersin K, Min YI, Meinert CL (1992). Factors influencing publication of research results. Follow-up of applications submitted to two institutional review boards. JAMA.

