# Supplementary material for: Development and validation of a recommended checklist for assessment of surgical videos quality: the LAParoscopic surgery Video Educational GuidelineS (LAP-VEGaS) video assessment tool
Source: Surg Endosc. 2020 Apr 6;35(3):1362–9. doi: 10.1007/s00464-020-07517-4 (PMC7886732; doi:10.1007/s00464-020-07517-4)
Supplement: Supplementary file 1 — Supplementary file1 (DOCX 18 kb) [file 464_2020_7517_MOESM1_ESM.docx]

Appendix 1. 37 LAP-VEGaS guidelines statements

- 1. **Author’s information and video introduction**

**S1. The video must include authors’ information such as names, Institution(s), country, year of surgery. Contact details of the corresponding author must be provided.**

**S2. It should be specified if the video was presented at national/international meetings or recorded during a live broadcast.**

**S3. The title of the video must include the name of the procedure performed and of the pathology treated.**

**S4. If the video is intended for training this should be specified and specific learning objectives could be presented. Aim of the video and relevance of the case presented should be stated.**

**S5. In case the procedure is performed by a surgical trainee it should be mentioned how many previous cases the trainee has performed in order to contextualise the video.**

**S6. Patient consent should be obtained.**

**S7. A conflict of interest disclosure must be present.**

- 1. **Case presentation**

**S8. All radiology pictures, videos and reports should be anonymised and the name of the patient should never be mentioned. All patient recognisable body parts such as eyes and tattoos should be obscured.**

**S9. The video should include one or more slides or audio-commentary with formal presentation of the case, including age, sex, American society of Anaesthesiologist score (ASA), body mass index (BMI), indication for surgery, comorbidities and history of previous surgery.**

**S10. Results of preoperative imaging should be presented.**

**S11. Pre-operative treatments, workup for surgery and blood test results should also be briefly presented if relevant for the case.**

- 1. **Demonstration of the surgical procedure**

**S12. The position of the patient on the operating table must be clearly demonstrated, including variations during the surgery.**

**S13. The position of the surgical and anaesthetic team should be demonstrated, including scrub nurse position and position of extra assistants.**

**S14. The position of the trocars must be detailed. It should be mentioned where additional trocars can be inserted in case of unexpected findings or technical difficulties.**

**S15. The site for specimen extraction should be demonstrated.**

**S16. Details of special equipment needed for the procedure should be provided, such as vessel sealer devices, wound protectors, manipulators and surgical staplers.**

**S17. The surgical procedure should be presented in a standardised step by step fashion.**

**S18. Every chapter should be clearly introduced and explained. The intraoperative findings need to be demonstrated, with constant reference to the anatomy.**

**S19. Additional manoeuvers and suggestions to face “progression failure” should be demonstrated – for instance, additional ports or assistants, change of the position of the patient or rescue manoeuvres in case of unexpected events such surgical stapler malfunction or equipment failure.**

**S20. Relevant additional intraoperative investigations should be mentioned and demonstrated.**

**S21. Describing the criteria for conversion to open surgery and the site of the incision in case of conversion might be useful in training videos.**

**S22. The open part of the procedure should be mentioned or demonstrated if the video is intended for training.**

- 1. **Outcomes of the procedure**

**S23. Outcomes of the procedure must be presented, including operating time, blood loss, cosmesis with picture of the healed wounds, length of hospital stay and postoperative morbidity.**

**S24. Histopathology assessment of the specimen should be presented. In case of malignancy number of retrieved lymph nodes and TNM staging should be detailed. Pictures of the specimen are desirable.**

- 1. **Associated educational content**

**S25. Additional educational content must be included. Diagrams, photos, snapshots and tables should be used to demonstrate anatomical landmarks, relevant or unexpected findings.**

**S26. Audio/written commentary in English language must be provided.**

- 1. **Peer review of surgical videos**

**S27. Educational videos must undergo formal peer review prior to publication. It should be stated if the video has been peer reviewed prior to publication.**

**S28. Peer review should assess not only the safety of the procedure performed, but also the supplementary educational content presented.**

**S29. Peer review should be undertaken by both surgical trainers and trainees.**

**S30. Videos should be amended and resubmitted, where possible, according to the reviewers’ comments with a point by point answer.**

**S31. Image quality should be assessed. When excessive smoke, low definition or suboptimal views are present for more than 25% of the duration of the procedure, the video should be rejected for poor image quality.**

**S32. Video should play at 1x speed. Where video is played at faster or slower the speed should be indicated in the respective video segments (e.g., 2x, 4x, 0.5x).**

- 1. **Use of surgical videos in educational curriculae**

**S33. Routine video-recording of the procedure and review with feedback sessions should be mandatory in every training program.**

**S34. Video recording can be useful for continue professional development even at the completion of the learning curve, in order to review unusual findings and to reflect on complications and outcomes.**

**S35. Videos demonstrating unusual cases and management of intraoperative complications should be shared at conferences.**

**S36. Formative assessment of the surgical performance should involve peer-review of unedited videos, using standardised assessment tools.**

**S37. The web platform should record the number of times the video has been watched for audit purposes. Moreover, it should allow comments and webchats in order to facilitate feedback and interaction amongst trainers and trainees.**
