# Supplementary material for: Development and validation of a recommended checklist for assessment of surgical videos quality: the LAParoscopic surgery Video Educational GuidelineS (LAP-VEGaS) video assessment tool
Source: Surg Endosc. 2020 Apr 6;35(3):1362–9. doi: 10.1007/s00464-020-07517-4 (PMC7886732; doi:10.1007/s00464-020-07517-4)
Supplement: Supplementary file 2 — Supplementary file2 (DOCX 19 kb) [file 464_2020_7517_MOESM2_ESM.docx]

**DELPHI PROCESS FOR DEVELOPMENT OF LAP-VEGAS MARKING SHEET**

For every item please answer the question **“This item should be included in the LAP-VEGaS marking sheet”**

| Nr | Item description | **1. Strongly disagree** | **2. Disagree** | **3. Neither agree or disagree** | **4. Agree** | **5. Strongly Agree** |
| --- | --- | --- | --- | --- | --- | --- |
| **1** | **Authors and Institution information. Title of the video including name of the procedure and pathology treated.** |  |  |  |  |  |
| **2** | **Formal presentation of the case, including age, sex, American society of Anaesthesiologist score (ASA), body mass index (BMI), indication for surgery, comorbidities and history of previous surgery. Anonymised relevant imaging is presented.** |  |  |  |  |  |
| **3** | **Position of patients, access ports, extraction site and surgical team** |  |  |  |  |  |
| **4** | **The surgical procedure is presented in a standardised step by step fashion.** |  |  |  |  |  |
| **5** | **The intraoperative findings are clearly demonstrated, with constant reference to the anatomy.** |  |  |  |  |  |
| **6** | **Relevant outcomes of the procedure are presented, including operating time, length of hospital stay and postoperative morbidity.** |  |  |  |  |  |
| **7** | **Histopathology assessment of the specimen is presented, supported by pictures of the specimen(s).** |  |  |  |  |  |
| **8** | **Additional educational content is included. (Diagrams, photos, snapshots and tables used to demonstrate anatomical landmarks, relevant or unexpected finding).** |  |  |  |  |  |
| **9** | **Audio/written commentary in English language is provided** |  |  |  |  |  |
| **10** | **The image quality is appropriate with constant clear view of the operating field and appropriate camera angle. Video speed is appropriate.** |  |  |  |  |  |
| **11** | **The video demonstrates an unusual case or management of intraoperative complications.** |  |  |  |  |  |
| **12** | **The procedure demonstrates competent use of dominant and nondominant hand with appropriate degree of traction and safe use of grasping and dissecting instruments** |  |  |  |  |  |
| **13** | **The procedure demonstrates appropriate speed and economy of movements, finishing one step before starting the next and avoiding rough tissue handling and unnecessary movements** |  |  |  |  |  |
| **14** | **The video is recorded full length or with minimal editing** |  |  |  |  |  |
